# Social Comparison and Stress Appraisal in Women with Chronic Illness

**DOI:** 10.3390/ijerph18105483

**Published:** 2021-05-20

**Authors:** M. Carmen Terol Cantero, Miguel Bernabé, Maite Martín-Aragón, Carolina Vázquez, Abraham P. Buunk

**Affiliations:** 1 Department of Behavioral Sciences and Health, University Miguel Hernández, 03202 Elche, Spain; macarmen@umh.es (M.C.T.C.); c.vazquez@umh.es (C.V.); 2 Department of Social and Organizational Psychology, Faculty of Psychology, National University of Distance Education, 28040 Madrid, Spain; mbernabe@psi.uned.es; 3 Honorary Academy Professor Evolutionary Social Psychology, University of Groningen, 9700 Groningen, The Netherlands; a.p.buunk@rug.nl

**Keywords:** social comparison, stress, women, chronic illness

## Abstract

(1) Background: The present study examined how social comparison orientation, stress appraisal and different social comparison strategies interact in women facing chronic illness. (2) Methods: Assessments were conducted by a trained professional in face-to face semistructured interviews (n = 179 women with chronic illness). Main outcome measures included social comparison scales and a stress appraisal questionnaire. The mediation model, by a bootstrapping procedure, was used to analyze the interaction among variables. (3) Results: Regarding the relationships among variables studied, they were related to each other except for a downward contrast, which allowed us to propose our hypothetical mediation model. Results showed that stress appraisal fully mediates between social comparison orientation and social comparison strategies except for the upward identification strategy. (4) Conclusions: Our results suggest that uncertainty, feelings of threat and low control over one’s illness or, in general, stress appraisal, had an important mediating effects over social comparison processes in patients with chronic illnesses. Therefore, by understanding the stress appraisal process, and the variables that might modify it, we could improve the use of social comparison as a favorable coping strategy.

## 1. Introduction

The Social Comparison Theory [1] proposes that a lack of information and uncertainty can trigger social comparison processes, and this is particularly common in the case of chronic illness [2,3,4,5,6]. Patients will try to search for relevant information and compare themselves with other patients with the same condition, such as the same illness diagnosis [3,7,8,9,10,11]. For instance, they may compare their symptoms or ways of coping in order to understand their chronic illness or to know how to adjust themselves to it [7,10,12,13,14]. The Identification-Contrast Model [15] establishes four different social comparison strategies for these patients: Upward Identification, Upward Contrast, Downward Identification and Downward Contrast. Patients may compare themselves with those who are in a better situation and are considered as similar (upward identification) or different (upward contrast). On the other hand, they may also compare themselves with those who are in a worse situation by focusing on differences (downward contrast) or similarities (downward identification). Many studies suggest that when people face threatening circumstances such as chronic pain or illness, they adopt such social comparison strategies as a way of coping with their illness [9,15,16,17].

According to Lazarus and Folkman’s model [18], social comparison strategies may act as a way of coping by focusing on emotion and/or the problem itself. Stress Appraisal (threat, uncertainty and lack of control) caused by chronic pain or illness is critical to understanding patients’ cognitive processes so that they can adapt to their illness. Stress Appraisal refers to the cognitive process activated by patients to assess their chronic illness as a stressful event, which exceeds their resources and that could have relevant consequences for well-being. For instance, there is evidence that the threat perceived by chronic patients leads to different comparison activities addressed to those who are less fortunate (downward evaluation), or even seeking information from more fortunate ones (upward contacts), as a means to try to cope and adjust themselves [14]. In this sense, positive and negative social comparisons can be treated as mediators of the relationships between stress appraisals (threat, uncertainty, harm or loss) and quality of life in the case patients with cancer [5]. Moreover, this tendency to compare with those who are doing better, and to contrast with those who are doing worse, was significantly associated with a dynamic stress appraisal and coping process, reinterpreting or changing their appraisal of the stressful event and the coping strategies to adapt themselves [19]. Specifically, in fibromyalgia patients, their illness impact was also explained by comparison strategies and the threat perceived (catastrophizing) by their chronic illness [20]. Social comparison processes may have effects on adjustment to chronic illness, but these effects depend on the individual differences and cognitive processes involved. Because of that, interventions focus on social comparison strategies that can have a positive effect on patients who experience higher stress appraisal (threat or uncertainty), or who perceive a negative health expectation, improving their resources for adaptation process and coping with illness impact. Not all patients obtain benefit from these interventions; for some chronic patients they can be harmful, so moderator analyses seem necessary to understand the interaction among these cognitive variables and to evaluate different social comparison interventions in chronic illness and in patients with cancer [21]. These findings demonstrate the importance of social comparisons in accounting for cognitive appraisals and how the interventions could improve quality of life and adjustment to chronic illness by reducing maladaptive comparisons that could be based on the patient’s stress appraisal [5].

In the context of Social Comparison theory and developments, social cognition perspective links social comparison processes to a wide range of theoretical and empirical approaches involved in social cognition research, considering different settings and individual characteristics [22]. In this work, we focus on a small part of all the issues that have received attention in the literature on social comparison and health. 

First, interest is placed on social comparison processes, taking into account the value of adding the stress model by Lazarus and Folkman [18] from a common social cognition perspective [17]. In this sense, Social Comparison and Stress Appraisal shared the hypothesis that uncertainty drives social comparison processes as ways of coping. Uncertainty is considered a characteristic of a stressful event and a dimension of stress appraisal (threat, uncertainty and lack of control). Stress appraisal refers to the process and assessment that patients make about the uncertainty and threat perceived by their chronic illness [18,22]. There is a long tradition in social comparison research including different stress, uncertainty and threat conditions [23,24]. But although stress appraisal is an important variable in the contribution of social comparison processes, specifically in the relationships proposed between this variable and social comparison processes, it has barely been explored in research studies [25,26]. In this sense, Arigo et al. [2] point out that individual differences such as social comparison orientation represent a promising avenue of research, and it is important to take into account moderating variables of social comparison processes in an integrative way. Therefore, this work attempts to support this line of research oriented to link cognitive processes to the variables developed from an integrative social cognition framework.

Second, theoretical and empirical studies focus on social comparison in health psychology in general, and in chronic illness specifically. So, three reasons justify the sample of women selected with diagnoses of Fibromyalgia and Breast Cancer: (1) the social comparison literature shows a majority of research publications in chronic illness conducted in cancer and rheumatology patients [2]; (2) both chronic illnesses cause an important uncertainty level related to diagnosis, symptoms, prognosis or treatment among other problems and (3) the prevalence of fibromyalgia diagnosis and breast cancer is higher in women, the preponderance of fibromyalgia or breast cancer in women versus men having an approximate ratio of 9:1 [27] and 100:1, respectively [28].

Finally, in the social comparison context, there are important individual differences in the extent to which people compare with others. In this sense, social comparison orientation refers to the personality disposition of individuals to compare with others. Therefore, it would seem important to include individual differences involved in this study such as Social Comparison Orientation. This is an important factor arising from research on the Social Comparison Theory, which also affects these cognitive processes. Social Comparison Orientation was proposed by Gibbons and Buunk [29] as a personality disposition of individuals who are prone to engaging in frequent social comparisons. In general, people who show high Social Comparison Orientation compare themselves more often with others and typically manifest a certain degree of self-uncertainty [29]. An increasing number of studies show how individuals with a high or low level in Social Comparison Orientation focus on different information and strategies of social comparison [7,13,30,31]. Nowadays, social comparison processes are considered an important research area in the analysis of different theoretical and empirical questions, including the different cognitive processes related to social comparison strategies [2,24].

Therefore, the aim of this study is to explore how Social Comparison Orientation, Stress Appraisal, and different social comparison strategies in women facing chronic illness (i.e., fibromyalgia, or a serious illness, i.e., cancer) interact in a mediation model. We analyze the relationships and structure of these variables in order to propose a hypothetical model of significantly associated cognitive processes. The model proposal is based on Social Comparison Orientation considered as an individual predisposition [29] and Stress Appraisal (uncertainty, threat and control) as the precursor of social comparison strategies [18,23,32,33]. Hence, our hypothesis is that Stress Appraisal is a mediator between Social Comparison Orientation and social comparison strategies.

## 2. Materials and Methods

### 2.1. Participants

The sample comprised 179 white Spanish women of the public health system (mean age: 51.87 ± 9.6), of which 24% were employed, 37.4% were housewives, 26.3% had temporary disability due to illness and 12.3% were unemployed or retired. Most were married or living with a partner, and 20.7% were single, divorced or widowed. With respect to educational level, the majority of the sample had skills in reading and writing or had completed primary (70.9%) and secondary education (20.7%). Eighty-nine of the participants were diagnosed with breast cancer, 68.8% of whom had had previous surgery and more than 80% were receiving chemotherapy and/or radiotherapy. The remaining 90 were fibromyalgia patients who were receiving different treatments for pain and/or anxiety and depression symptoms.

The inclusion criteria were: (1) fibromyalgia diagnosis confirmed by the American College of Rheumatology (ACR) criteria (Wolfe et al., 2010), (2) age over 18 years, (3) no previous psychiatric diagnosis and (4) ability to understand questionnaires.

### 2.2. Measures

Sociodemographic and Clinical Variables. We collected information about age, gender, employment situation, marital status, educational level, diagnosis and treatments.

Social comparison orientation. This was measured by the validated Spanish version of the Iowa-Netherlands comparison orientation measure [INCOM; [29]. It includes 11 items [INCOME-E; [10] with a 5-point Likert-type response scale ranging from 1 (strongly disagree) to 5 (strongly agree). Sample items are: ‘I often compare myself with others with respect to what I have achieved in life’ or ‘If I want to find out how well I have done something, I compare what I have done with how others have done’. Higher scores show higher levels of social comparison orientation. In the current sample, α = 0.78.

Stress appraisal. This was assessed by the Spanish adaptation of the Appraisal of the Stressor Scale [22], which was adapted by Terol et al. [34]. The final version for the Spanish sample includes an 11 item-scale measuring threat, uncertainty and control, using a 6-point Likert-type response scale (1 = strongly disagree; 6 = strongly agree). The scale begins with the stem “in general my problem (chronic illness”, followed by items such as “is very threatening”, “its course is unknown” or “is out of control”. In a total score of stress appraisal, high scores show greater uncertainty, threat and lower control. In this sample α = 0.82.

Social comparison strategies. These were measured by the Social Comparison in Illness Scale [19] Spanish adapted by Terol et al. [35]. It includes 12 items with a 5-point Likert-type response scale (1 = never; 5 = very often). Three items were included (range: 3–15) for each of the four subscales of social comparison strategies: upward identification, upward contrast, downward identification and downward contrast. Examples of an item for each subscale are: “When I think about or see others who are better off than I am, I am very hopeful that my situation will improve” (upward identification); “When I think about or see others who are better off than I am, it is threatening to notice that I am not doing so well” (upward contrast); “When I think about or see others who are worse off than I am, I feel afraid that my health will decline” (downward identification); “When I think about or see others who are worse off than I am, I realize how well I am doing” (downward contrast); Cronbach’s alphas for each of the subscales of social comparison strategies are upward identification, α = 0.97; upward contrast, α = 0.97; downward identification, α = 0.89; and downward contrast α = 0.91. Higher scores show a greater frequency of comparison strategies.

### 2.3. Procedure

This was a cross-sectional study with a nonprobability convenience sample. After the Ethics Committee’s approval of the study, health professionals from the Oncology and Rheumatology Departments at different hospitals in Alicante (Spain) selected participants from the outpatients. The patients were informed about the aim of the study and the possibility of voluntary participation in the study. Once the patient agreed, a trained professional asked for the patient’s consent and they signed a commitment to participate. The questionnaires were administered in a semistructured interview. In this study, the final sample size and its features are equivalent to those used in other relevant social comparison research studies on chronic illness reviewed by Arigo et al. [2].

This study was conducted in accordance with the Declaration of Helsinki. Data collection was carried out under the FISABIO agreement (FISHOSVI1.16D/2013–2019). The specific study sample was collected for three years involving several hospitals from Alicante (SPAIN).

### 2.4. Data Analysis

IBM SPSS v.22 software (IBM Corp, Armonk, NY, USA) was used for the statistical analysis. Analyses of the scales’ internal consistency (Cronbach’s alpha) were carried out to establish their validity. Values above α = 0.70 indicated a good level of internal consistency. Although alpha = 0.80 is recommended, different authors support that alpha > 0.70 can be considered “acceptable” or “satisfactory” [36,37] when taking into account other characteristics such as number of items on the scale or type of research (early stage, exploratory).

Descriptive analyses (mean and Pearson correlation matrix) were carried out. Stress and Social Comparison as independent variables showed normal distributions. Parametric statistics should be performed when sample size is above 20 responses, and the statistical hypothesis is based on the mean. In addition, parametric tests are more powerful to contrast null hypotheses and, while Likert scale represents an underlying continuous variable, the individual element analyses in exploratory studies should be performed with parametric tests [38].

We performed an iterative K-means cluster analysis (nonhierarchical method; K = 2) to reflect the structure or the relationships among variables, and differences in the cluster solution were analyzed by Analyze of Variance (ANOVA) (F-Fisher with *p* < 0.05 was accepted). Cluster analysis for group observations was chosen because it is: (1) an exploratory statistical technique which allows group observations (people, things, events) with strong degree of association or structure between them in the same cluster, reducing the number of variables by grouping them, (2) less restrictive in its assumption regarding sample or variable characteristics and (3) a key for identifying relationships in an effective manner between groups by recognizing patterns or profiles. Moreover, in health psychology, cluster analysis approaches both theoretical and practical problems, thus closing the gap between the variables and the individual problems. Therefore, cluster analysis provides a major contribution applied in health psychology research by identifying groups that are oriented towards one or other interventions or further research in this line [39]. Prior to clustering, multicollinearity was tested according to VIFs < 10 [40]. This is a limit score as stated in the literature, but some authors confirm that VIFs values between 5 to <10 show multicollinearity problems so that values <2.5 are always recommended [40]. Regarding the ANOVA, it was obtained by taking the groups defined by the clusters as a factor, and each of the variables included in the analysis as a dependent variable. This was an implicit process in the calculation of the groups, so if the solution was a single factor, the ANOVA would not be shown. The discriminant function was also explored through contingency tables and χ^2^ statistics in order to validate the cluster’s structure [39].

In the mediation analysis, Preacher and Hayes’s [41] bootstrapping procedure was used to analyze the indirect effect on the dependent variable. According to these authors, indirect effects are significant when *p* < 0.05 with a 95 percent confidence interval (CI). To determine the strength of mediation, the SPSS macro developed by Preacher, Rucker and Hayes [42] was used. This procedure performs multiple regression analyses in order to test indirect and direct effects on the dependent variable. In this study, analyses were applied according to Model 4 [42], which operates as Simple Mediation Analysis with a unique mediator, and the mediating effect was tested using Sobel’s test. To establish the mediating effect on the dependent variable, the corresponding critical value for α = 0.05 is z/2 = 1.96. Disease was identified as a control variable.

## 3. Results

### 3.1. Relationships and Structure: Social Comparison Orientation, Stress Appraisal and Social Comparison Strategies

Table 1 shows means, standard deviations, range scores and correlations among all the study variables. The social comparison orientation mean score was above 3.0 points (SD = 0.73) and the stress appraisal mean score was 4.07 (SD = 1.03). For social comparison strategies, upward identification and downward contrast showed higher mean scores of 3.29 and 3.03, respectively. Social comparison orientation correlated with stress appraisal (r = 0.25 *p* < 0.01), upward contrast and downward identification. In addition, stress appraisal also showed negative correlations with upward identification (*p* < 0.01) and positive correlations with upward contrast and downward identification strategies. However, downward contrast showed no relation with social comparison orientation or stress appraisal.

As shown in Table 2, we performed K-means cluster analysis and assessed differences using ANOVA to identify relations within the variables studied (social comparison orientation with stress appraisal and social comparison strategies; identification and contrast). Multicollinearity was measured by VIFs < 2. We identified two patterns of relationships: the first pattern was characterized by a lower social comparison orientation (*p* < 0.01) with lower stress appraisal and more use of upward identification (*p* < 0.001); the second pattern was characterized by a higher social comparison orientation (*p* < 0.01) with higher stress appraisal and more use of upward contrast and downward identification strategies (*p* < 0.001). The downward contrast strategy did not show any significant differences between cluster results.

With respect to lower or higher levels of social comparison orientation (<3 or >3) and stress appraisal (<4 or >4) (see Table 3), 57.8% lower comparers and 55.43% higher comparers were classified in cluster 1 and 2, respectively (χ^2^ = 0.054). However, stress appraisals of 89.61% (lower level) and 79.59% (higher level) classified patients in cluster 1 and 2, respectively (χ^2^ = 82.62; *p* ≤ 0.001).

The results presented thus far show that, except for downward contrast, most of the variables were related to each other, which allowed us to propose a mediation model with the rest of the variables studied according to Baron & Kenny’s [43] criteria and conditions.

### 3.2. A Hypothetical Model: Stress Appraisal as a Mediator between SCO and SC Strategies

The effects obtained from social comparison orientation for the different strategies in the mediational analyses performed can be seen in Table 4. In the first step, social comparison orientation predicted stress appraisal in the sample analyses (R^2^ = 0.58, F = 45.88, *p* = 0.000), and it was also observed to have an effect on it (β = 0.13, SE = 0.06, *p* = 0.012). For total effects, all variables showed a significant *p*-value (*p* < 0.05), while direct effects (See Table 4) showed that stress appraisal fully mediated between social comparison orientation and social comparison strategies (all *p*’s > 0.05) except for the upward identification strategy (*p* = 0.002). However, a partial mediation can be observed in upward identification strategy (β = 0.20, SE = 0.14, *p* = 0.003), where the possible effect of disease was controlled in the regression but there was no covariance effect (all *p*’s > 0.05).

**Figure 1:**
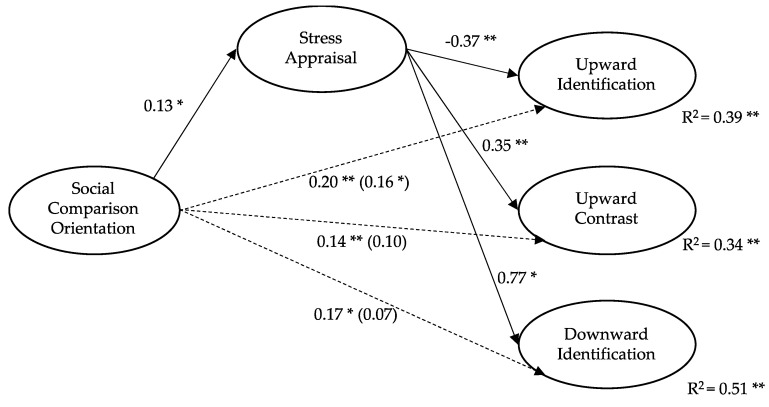
Regression model of social comparison strategies (Note: * *p* < 0.05; ** *p* < 0.01).

The indirect effects observed show that social comparison orientation through stress appraisal had a significant impact on upward identification (β = −0.05, SE = 0.02, CI [−0.106–−0.013], upward contrast (β = 0.06, SE = 0.03, CI [0.015–0.120]) and downward identification strategies (β = 0.10, SE = 0.04, CI [0.039–0.172]). Sobel’s test showed higher values according to the criterion (z/2 > 1.96, *p* < 0.05) for all indirect effects observed. Specifically, these values were for upward identification (z/2 = −2.00, *p* = 0.05), upward contrast (z/2 = 2.14, *p* = 0.03) and downward identification strategies (z/2 = 2.51, *p* = 0.01). Figure 1 shows the proposed regression model for these social comparison strategies.

## 4. Discussion

The aim of this study was to explore the relationships of social comparison orientation and social comparison strategies with stress appraisal in a hypothetical mediation model. Patients were women with chronic illnesses; specifically, they were diagnosed with breast cancer or fibromyalgia. First of all, related to the preliminary results, we found that, on average, stress appraisal was relatively high, and that upward identification and downward contrasts were the social comparison strategies used most by these patients. These results suggest that social comparison is fairly regular among patients facing a health threat. Patients tend to engage in social comparison that focuses on others who are better off than themselves in order to identify with them, and on the differences with others who are worse off in order to maintain or attain a high level of well-being. These results are similar to those reported in other studies in patients with chronic illness who use these positive or favorable social comparison strategies more frequently [4,44].

With respect to the relationships between the variables studied, we found that social comparison orientation correlated positively with stress appraisal, upward contrast and downward identification. In addition, stress appraisal showed correlations with three social comparison strategies; negatively with upward identification, and positively with upward contrast and downward identification. These results were complemented by two patterns of relationships: (1) a low social comparison orientation and stress appraisal associated with more upward identification, and (2) a high social comparison orientation and stress appraisal associated with more upward contrast and downward identification. However, downward contrast did not correlate with social comparison orientation or stress appraisal and did not show significant differences between these two patterns of relationships. As in previous research, this pattern of relationships showed how patients with higher social comparison orientation experienced relatively more uncertainty and stress, and used different and more “unfavorable” social comparison strategies than patients with lower social comparison orientation [13,30]. However, we found that stress appraisal discriminated the profiles or relationship patterns better than social comparison orientation; 89.61% and 79.59% with lower and higher stress appraisal were classified in cluster 1 and 2.

Second, we confirmed the mediation effect of stress appraisal by explaining the frequency of certain social comparison strategies used (upward contrast, downward identification and upward identification), and results were consistent with the literature focusing on how these types of variables interact simultaneously [21]. These results suggest that uncertainty as a dimension of stress appraisal of one’s illness may determine social comparison processes as a coping strategies used by patients with chronic illnesses. These results may reflect possible relationships that allow us to further understand the role of each cognitive construct frequently assessed in chronic illness. As in other studies, our results also highlight how threat, uncertainty and stress seem to establish the strongest relationships with “unfavorable” social comparison strategies (upward contrast, downward identification). This underlines their special relevance in an adaptation and adjustment processes or intervention for these patients [2,5,10,13,21,45]. Finally, the partial mediational effect of stress appraisal on upward identification may indicate that these “favorable” strategies can behave in a different way. They could establish direct and/or mediate relationships that have a greater association with other personal variables such as positive thinking strategies and social support resources, or specific illness features related to perceived symptoms or prognosis [5,20]. In sum, patients high Social Comparison Orientation mediated by high stress appraisal mobilize more “unfavorable” strategies and they have more difficulties in identifying themselves with others who are considered to be in a better situation. Therefore, by understanding the stress appraisal process and the variables that might modify it, we could improve the use of social comparison as a favorable coping strategy. Patients that use cognitive resources to adjust primary and secondary stress appraisal could be useful as “referents” or “models” before the illness becomes severe, which could also facilitate more favorable or adaptive coping strategies for achieving a better adaptation to chronic illness.

### Limitations

The first limitation of this study is that all the participants were female. However, among breast cancer and fibromyalgia patients, women are overrepresented. Second, as the patients were selected on the basis of their accessibility, we considered homogeneous convenience sampling criteria; sociodemographic or clinical factors of the general population [46]. In this sense, we verified that our sample features were similar to those found in other studies on FM and breast cancer patients.

Next, we did not test the mediating effect in patient subgroups, i.e., higher vs. lower stress perceived, type of comparers or other variables. An additional limitation is that it was a cross-sectional study while longitudinal studies could further and better clarify the role of the variables. Moreover, the use of semistructured interviews and self-reported measures can introduce social desirability bias by interviewer presence, and also the patients may be forced to answer or choose among several closed replies.

Finally, we think that future studies on social comparison and cognitive processes should be able to provide an even more detailed analysis of the different patterns of relationships by taking into account other variables involved, such as features or stage of illness (time since diagnosis, prognosis, severity of symptoms), context or settings (hospital, primary health care level, self-help or network groups), the stress appraisal process at different stages (harm, loss, threat, uncertainty) and other personal variables (optimism, self-esteem, neuroticism or social support resources) [5,6,21].

## 5. Conclusions

The results of this study suggest that uncertainty, feelings of threat and low control over one’s illness or, in general, stress appraisal, had an important mediating effect on social comparison processes in patients with chronic illnesses. Therefore, by understanding the stress appraisal process and the variables that might modify it, we could improve the use of social comparison as a favorable coping strategy.

In this sense, this work contributes to theoretical and practical of social comparison field in two ways. (1) It shows an approach for understanding different cognitive processes in an integrative social cognition perspective with stress appraisal variable involved. This allows us to approach the dynamic and complexity of cognitive processes under specific circumstances of uncertainty and threat caused by a chronic disease. (2) As practical implications, our results suggest the importance in moving from “negative” stress appraisal (more uncertainty and threat perceived) toward more “positive” reappraisals, including upward identification and downward contrast such as resources and strategies to use for coping with chronic illness and reaching better adjustment [2,4,20]. This is especially relevant for patients with high social comparison orientation who are more inclined to use different social comparison strategies more frequently and who experience relatively more uncertainty. Therefore, from health settings, professionals can minimize the patient’s uncertainty through adequate information to provide control and self-efficacy perception to cope with chronic illness.

## Figures and Tables

**Table 1:** Descriptive Analyses. Means and Pearson correlation matrix.

	Mean	SD	1	2	3	4	5	6
1. Social comparison orientation	3.03	0.73		0.25 **	0.13	0.19 *	0.18 *	0.14
2. Stress appraisal	4.07	1.03			−0.32 **	0.32 **	0.47 **	−0.10
3. Upward identification strategy	3.29	1.54				−0.14	−0.14	0.40 **
4. Upward contrast strategy	2.98	1.48					0.34 **	0.13
5. Downward identification strategy	2.31	1.31						0.02
6. Downward contrast strategy	3.03	1.35						

Note: * *p* < 0.05; ** *p* < 0.01.

**Table 2:** K-Means clusters for social comparison orientation, stress appraisal and social comparison strategies (identification, contrast). Mean differences (ANOVA).

	Cluster 1	Cluster 2	F-Fisher	*p*-Value
	(n = 89)	(n = 86)		
1. Social comparison orientation	2.87 (0.81)	3.20 (0.62)	9.02	**
2. Stress appraisal	3.37 (0.87)	4.76 (0.63)	145.79	***
3. Upward identification strategy	3.99 (1.31)	2.57(1.40)	48.24	***
4. Upward contrast strategy	1.71 (1.01)	2.93 (1.31)	47.87	***
5. Downward identification strategy	1.96 (1.10)	4.07 (0.94)	182.53	***
6. Downward contrast strategy	3.12 (1.38)	2.93 (1.29)	0.92	n.s.

Note: ** *p* < 0.01; *** *p* < 0.001; n.s. = non-significant.

**Table 3:** Contingency table analysis and Chi-Square Test. Cluster 1, 2 according to lower-higher level of social comparison orientation and stress appraisal.

	SCO− (n = 83)	SCO+ (n = 92)	SA− (n = 77)	SA+ (n = 98)
Cluster 1. (n = 89)	57.8%	44.57%	89.61%	20.41%
Cluster 2. (n = 86)	42.2%	55.43%	10.39%	79.59%
	100%	100%	100%	100%
	χ^2^ = 0.054 (n.s.)		χ^2^ = 82.62 ***	

Note: SCO− = Social Comparison Orientation < 3; SCO+ = Social Comparison Orientation > 3; SA− = Stress Appraisal < 4; SA+ = Stress Appraisal > 4; χ^2^ = Chi-square; *** *p* < 0.001; n.s. = non-significant.

**Table 4:** Total and direct effects of social comparison strategies.

	β	SE	*p*-Value	*t*	R^2^	ΔR^2^	F
Total Effects							
Upward identification strategy	0.20	0.15	0.002	2.27	0.31		9.08 *
Upward contrast strategy	0.14	0.13	0.024	2.27	0.23		4.68 *
Downward identification strategy	0.17	0.15	0.039	2.07	0.23		4.88 *
Direct Effects							
Upward identification strategy	0.16	0.15	0.003	2.97	0.39	0.08	10.47 *
Upward contrast strategy	0.10	0.13	0.122	1.55	0.34	0.11	7.94 *
Downward identification strategy	0.07	0.13	0.368	0.90	0.51	0.28	19.78 *

Control variable: Illness (cancer and fibromyalgia) (all *p’s* > 0.05)/* *p* < 0.05.

## Data Availability

The data presented in this study are available on request from the corresponding author.

## References

[1] Festinger L.A. (1954). Theory of Social Comparison Processes. Hum. Relat..

[2] Arigo D., Suls J., Smyth J.M. (2012). Social Comparisons and Chronic Illness: Research Synthesis and Clinical Implications. Health Psychol. Rev..

[3] Stiegelis H.E., Hagedoorn M., Sanderman R., Bennenbroek F.T., Buunk A.P., Van den Bergh A.C., Botke G., Ranchor A.V. (2004). The Impact of an Informational Self-Management Intervention on the Association between Control and Illness Uncertainty before and Psychological Distress after Radiotherapy. PsychoOncology.

[4] Terol M.C., Neipp M.C., Lledó A., Pons N., Bernabé M. (2012). Comparación Social y Variables Psicosociales Relacionadas: Una Revisión de Cáncer y Dolor Crónico. An. Psicol..

[5] Umstead K.L., Kalia S.S., Madeo A.C., Erby L.H., Blank T.O., Visvanathan K., Roter D.L. (2018). Social Comparisons and Quality of Life Following a Prostate Cancer Diagnosis. J. Psychosoc. Oncol..

[6] Buunk A.P., Bennenbroek F., Stiegelis H.E., Van den Bergh A.C., Sanderman R., Hagedoorn M. (2011). Follow-up Effects of Social Comparison Information on the Quality of Life of Cancer Patients: The Moderating Role of Social Comparison Orientation. Psychol. Health.

[7] Buunk A.P., Belmonte J., Peiró J.M., Zurriaga R., Gibbons F.X. (2005). Diferencias Individuales En La Comparación Social: Propiedades de La Escala Española de Orientación Hacia La Comparación Social. Rev. Latinoam. Psicol..

[8] Corcoran K., Cruisius J., Mussweiler T., Chadee D. (2011). Social Comparison: Motives, standards and mechanisms. Theories in Social Psychology.

[9] Tennen H., McKee T.E., Affleck G., Suls J.M., Wheeler L. (2000). Social comparison processes in health and illness. Handbook of Social Comparison: Theory and Research.

[10] Terol M.C., Buunk A.P., Cabrera V., Bernabé M., Martin-Aragón M. (2020). Profiles of Women with Fibromyalgia and Social Comparison Processes. Front. Psychol..

[11] Cabrera V., Buunk A.P., Terol M.C., Quiles Y., Martin-Aragón M. (2017). Social Comparison Processes and Catastrophising in Fibromyalgia: A Path Analysis. Psychol. Health.

[12] Butzer B., Kuiper N.A. (2006). Relationships between the Frequency of Social Comparisons and Self-Concept Clarity, Intolerance of Uncertainty, Anxiety, and Depression. Personal. Individ. Differ..

[13] Buunk A.P., Zurriaga R., González P., Terol M.C., López-Roig S. (2006). Targets and Dimensions of Social Comparison among People with Spinal Cord Injury and Other Health Problems. Br. J. Health Psychol..

[14] Taylor S.E., Lobel M. (1989). Social Comparison Activity under Threat: Downward Evaluation and Upward Contacts. Psychol. Rev..

[15] Buunk A.P., Ybema J.F., Buunk B.P., Gibbons F.X. (1997). Social comparisons and occupational stress: The identification-contrast model. Health, Coping, and Well-Being: Perspectives from Social Comparison Theory.

[16] Wood J.V., VanderZee K., Buunk B.P., Gibbons F.X. (1997). Social comparisons among cancer patients: Under what conditions are comparisons upward and downward?. Health, Coping, and Well-Being: Perspectives from Social Comparison Theory.

[17] Buunk A.P., Gibbons F.X. (1997). Health, Coping, and Well-Being: Perspectives from Social Comparison Theory.

[18] Lazarus R.S., Folkman S. (1984). Stress, Appraisal, and Coping.

[19] Van der Zee K., Buunk B., Sanderman R., Botke G., Van den Bergh F. (2000). Social Comparison and Coping with Cancer Treatment. Personal. Individ. Differ..

[20] Suls J., Martin R., Wheeler L. (2002). Social comparison: Why, with whom, and with what effect?. Curr. Dir. Psychol. Sci..

[21] Brakel T.M., Dijkstra A., Buunk A.P. (2014). Targeting Cancer Patients’ Quality of Life through Social Comparison: A Randomised Trial. Psychol. Health.

[22] Vitaliano P.P., Russo J., Weber L., Celum C. (1993). The Dimensions of Stress Scale: Psychometric Properties1. J. Appl. Soc. Psychol..

[23] Gibbons F.X., Gerrard M., Suls J., Wills T.A. (1991). Downward comparison and coping with threat. Social Comparison: Contemporary Theory and Research.

[24] Gerber J.P., Wheeler L., Suls J. (2018). A Social Comparison Theory Meta-Analysis 60+ Years On. Psychol. Bull..

[25] Croyle R.T. (1992). Appraisal of health threats: Cognition, motivation, and social comparison. Cogn. Ther. Res..

[26] Osterman L.M.R. (1996). Stress Appraisal: The Role of Social Comparison.

[27] Katz J.D., Mamyrova G., Guzhva O., Furmark L. (2010). Gender bias in diagnosing fibromyalgia. Gender Med..

[28] International Agency for Research on Cancer. World Health Organization Global Cancer Observatory. https://gco.iarc.fr/.

[29] Gibbons F.X., Buunk A.P. (1999). Individual Differences in Social Comparison: Development of a Scale of Social Comparison Orientation. J. Pers. Soc. Psychol..

[30] Buunk A.P., Dijkstra P., Bosch Z.A., Dijkstra A., Barelds D.P.H. (2012). Social Comparison Orientation as Related to Two Types of Closeness. J. Res. Personal..

[31] Buunk B.P., Oldersma F.L., de Dreu C.K.W. (2001). Enhancing Satisfaction through Downward Comparison: The Role of Relational Discontent and Individual Differences in Social Comparison Orientation. J. Exp. Soc. Psychol..

[32] Galvin L.R., Godfrey H.P. (2001). The Impact of Coping on Emotional Adjustment to Spinal Cord Injury (SCI): Review of the Literature and Application of a Stress Appraisal and Coping Formulation. Spinal Cord.

[33] Buunk B.P., Brenninkmeijer V. (2001). When Individuals Dislike Exposure to an Actively Coping Role Model: Mood Change as Related to Depression and Social Comparison Orientation. Eur. J. Soc. Psychol..

[34] Terol M.C., Quiles Y., Pérez V. (2012). Manual de Evaluación Psicosocial en Contextos de Salud.

[35] Terol C., Lledó A., Quiles Y., Martín-Aragón M. (2015). Adaptation and Validation of the Spanish Version of the Social Comparison Scale in Chronic Illness Patients. J. Health Psychol..

[36] Nunnally J.C., Bernstein I.H. (1994). The Assessment of Reliability.

[37] Frias-Navarro D. (2020). Apuntes de Consistencia Interna de las Puntuaciones de un Instrumento de Medida.

[38] Jaimeson S. (2005). Likert Scales: How to (ab) Use Them. Med. Educ..

[39] Clatworthy J., Buick D., Hankins M., Weinman J., Horne R. (2005). The Use and Reporting of Cluster Analysis in Health Psychology: A Review. Br. J. Health Psychol..

[40] Kutner M.H., Nachstheim C.J., Neter J. (2004). Applied Linear Regression Models.

[41] Preacher K.J., Hayes A.F. (2004). SPSS and SAS Procedures for Estimating Indirect Effects in Simple Mediation Models. Behav. Res. Methods Instrum. Comput..

[42] Preacher K.J., Rucker D.D., Hayes A.F. (2007). Addressing Moderated Mediation Hypotheses: Theory, Methods, and Prescriptions. Multivar. Behav. Res..

[43] Baron R.M., Kenny D.A. (1986). The Moderator–Mediator Variable Distinction in Social Psychological Research: Conceptual, Strategic, and Statistical Considerations. J. Pers. Soc. Psychol..

[44] Dibb B., Yardley L. (2006). Factors Important for the Measurement of Social Comparison in Chronic Illness: A Mixed-Methods Study. Chronic Illn..

[45] Bouchard L.C., Fisher H.M., Carver C.S., Kim Y., Antoni M.H. (2019). Social Comparisons Predict Health-Related Quality of Life and Depressive Symptoms across the First Year of Breast Cancer Treatment. Psychooncology.

[46] Jager J., Putnick D.L., Bornstein M.H. (2017). More than Just Convenient: The Scientific Merits of Homogeneous Convenience Samples. Monogr. Soc. Res. Child Dev..

